# Latex-Free Anesthesia for Craniosynostosis Surgery Associated With Xia-Gibbs Syndrome: A Case Report

**DOI:** 10.7759/cureus.46544

**Published:** 2023-10-05

**Authors:** Matheus S Nascimento, Sarah G de Paula, Thiago C Lago Alves, Bruna G Noronha, Heitor Medeiros

**Affiliations:** 1 Department of Anaesthesiology, Hospital Universitário Onofre Lopes, Natal, BRA; 2 Department of Medicine, Universidade Potiguar, Natal, BRA; 3 Department of Anaesthesiology, Secretaria Municipal da Saúde (SMS) de Riberão Preto, Ribeirão Preto, BRA

**Keywords:** total cranial vault remodelling, pediatric anesthesiology, anesthetic challenges, bleeding risk, rare genetic mutation, latex-fruit syndrome, xia-gibbs syndrome, craniosynostosis

## Abstract

This case report describes the anesthetic management of a one-year-old patient with Xia-Gibbs syndrome, which is a rare genetic condition caused by a mutation in the *AHDC1* gene. The procedure involved calvarial vault remodeling and fronto-orbital advancement to correct a left coronal craniosynostosis. In addition, the patient had a history of seizures and latex-fruit syndrome, which necessitated careful preoperative planning and management. Despite the difficulties provided by the patient's cranial abnormalities and the paucity of literature on anesthetic experiences with the condition, the treatment was completed successfully and without complications. Insights are offered about the anesthetic approach for this syndromic pediatric patient undergoing neurosurgery with a high risk of bleeding. It is important to understand and prepare for the perioperative implications of this disease in order to achieve a safe outcome.

## Introduction

Xia-Gibbs syndrome (XGS), first described in 2014, is an autosomal dominant genetic condition associated with a de novo mutation in the *AHDC1* gene [1]. There are 390 confirmed cases to date, but it is estimated that there should be around 100,000 non-diagnosed [2]. In this particular case, XGS was related to latex allergy, a rare disorder that affects approximately 1-2% of the global population and can be associated with multiple congenital anomalies [3].

This report describes the case of a one-year-old patient diagnosed with XGS who needed a surgical correction for craniosynostosis; all equipment used during the procedure was latex-free. While managing the airway was a concern due to cranial deformities, understanding and preparing for the broader health implications of this rare syndrome was crucial. This case report provides a straightforward look into our anesthetic approach, focusing on the decisions made and strategies used in the dearth of prior references, all aiming for a safe surgical outcome.

## Case presentation

**Figure 1:**
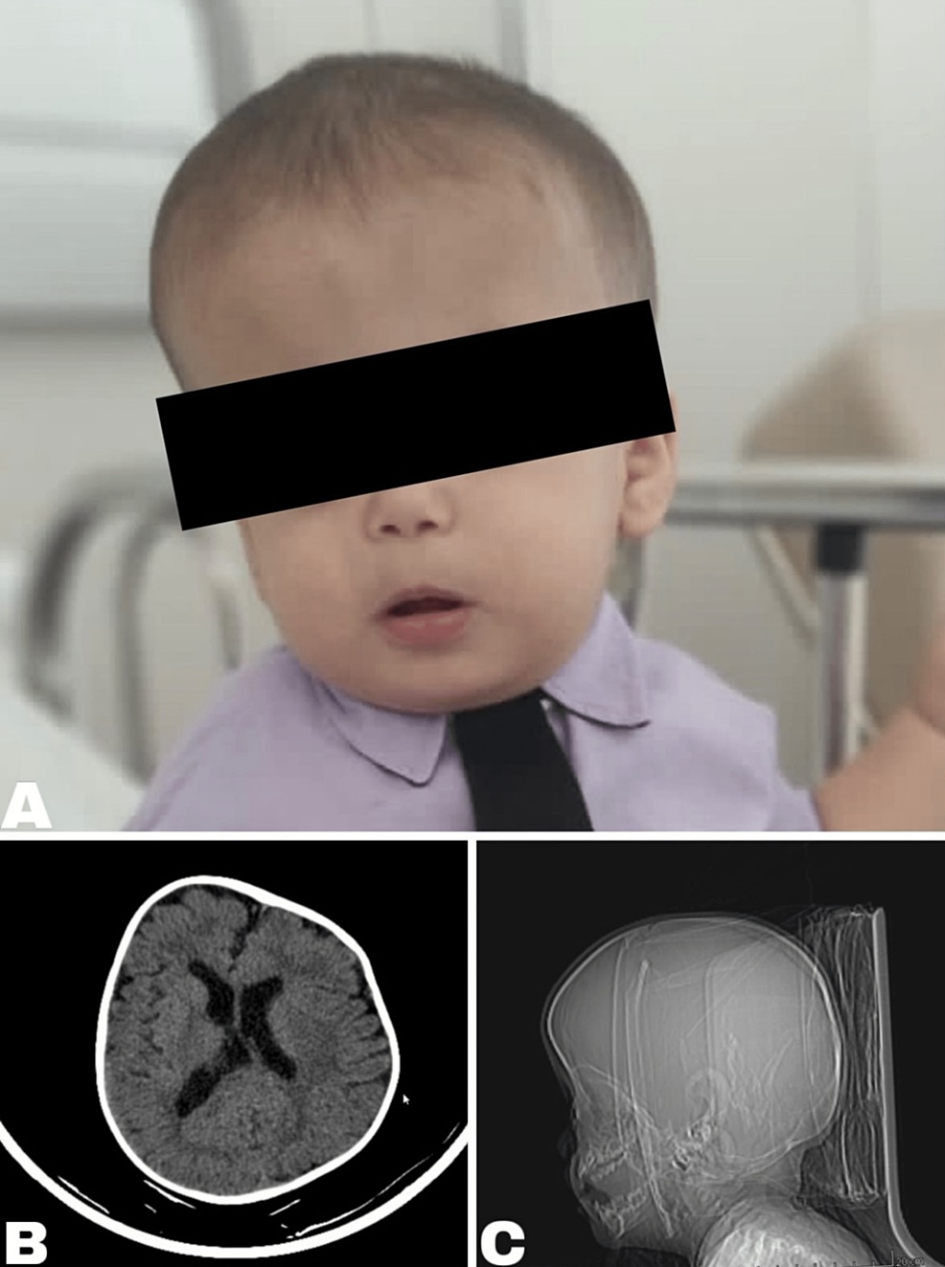
Our patient with Xia-Gibbs syndrome and his dysmorphic features A notable right frontal bulge seen in the (A) frontal view of the child with XGS, and in two tomographic slices, the axial (B) and the sagittal (C)

A 13-month-old male infant weighing 9 kg, with a height of 69 cm and BMI of 18,91 kg/m², had a genome containing a pathogenic variant in heterozygosis of the *AHDC1* gene, associated with XGS. There was phenotypical compatibility, with clinical findings such as delayed development, asymmetrical skull, bulging right forehead (Figure 1), pectus excavatum, depressed nasal base, and ogival palate, as well as complex congenital craniosynostosis. In addition, he had a pineapple and latex allergy, confirmed by an allergologist, as well as a past history of seizures. He was admitted to a tertiary hospital for his first surgery, a programmed elective surgical correction of a left coronal craniosynostosis. So, calvarial vault remodeling with fronto-orbital advancement was indicated.

The day before the surgery, the operating room was cleaned and isolated from all latex sources. According to our institutional protocol, it was the first surgery of the day in a signalized room with a restricted flow of people. After preparations, the patient was brought directly to the room with an allergy bracelet. He already had a right internal femoral central line. He was monitored with cardioscopy, peripheral oximetry, and invasive arterial blood pressure through the right dorsalis pedis artery with auxiliary ultrasound.

The patient’s initial vital signs were a heart rate of 111 beats per minute, a respiratory rate of 25 breaths per minute, oxygen saturation of 98%, systolic blood pressure of 94, and diastolic blood pressure of 65. He was pre-oxygenated for five minutes with 100% oxygen at 8 L/minute through a facial mask. Intravenous (IV) induction was realized with fentanyl at 2 mcg/kg, followed by 1 mg/kg lidocaine, 2 mg/kg propofol, 0.5 mg/kg esketamine, and 0.5 mg/kg rocuronium. Intubation was performed without complications by direct laryngoscopy with a Macintosh blade, size 0, and a cuffed 3.5 orotracheal tube. There was a perceivable ogival palate with a 2A Cormack-Lehane, but it was not considered a difficult airway.

General anesthesia was maintained with 2.8% sevoflurane and remifentanil, 0.1 mcg/kg/minute, IV. The patient was maintained in a supine position with a head-up tilt. Then, an indwelling bladder catheter was introduced for diuresis monitoring. Right after, a nasopharyngeal thermometer was added, as well as a full-body warming blanket.

Antifibrinolytic therapy was necessary due to the risk of bleeding. As our patient had seizures in the past, aminocaproic acid was chosen because it did not raise the risk, unlike tranexamic acid [4]. We administered an IV bolus of 100 mg/kg, followed by a continuous IV infusion of 40 mg/kg/hour [5]. Postoperative nausea and vomiting prophylaxis was realized with 1 mg IV each of dexamethasone and ondansetron, respectively, at the start and 30 minutes before the end of the surgery.

The anesthesia duration was five hours. Volemic reposition was realized with ringer lactate, correcting 10 hours of fasting. Also, the maintenance infusion rate was 40 mL/hour, guided by Holliday-Segar’s rule, totaling 350 mL throughout the surgery [6]. Also, as it was a calvarial vault remodeling, we decided to initiate 4 mL/kg of red blood cell concentrate around incision time to keep up with the ongoing losses, followed by an additional 5 mL/kg based on gasometry and losses, completing a total of 90 mL. We estimate around 200 mL of blood loss during surgery. The initial hemoglobin value was 11.8 g/dL, and the final value was 10.5 g/dL. Episodes of hypotension occurred and needed to be corrected with boluses of volume associated with aliquots of 5 mcg epinephrine IV. At the end, the total hydric balance was approximately zero.

Finally, morphine 20 mcg/kg was infused IV for postoperative pain control. Then, neuromuscular blockade was reversed with 2 mg/kg IV of sugammadex. The infant was extubated at the operation room and delivered at our pediatric intensive care unit, stable and without pain, respiratory disturbances, or vomiting episodes.

## Discussion

XGS mutated gene, *AHDC1*, is responsible for encoding a transcription factor for binding of deoxyribonucleic acid and is expressed in the mouse brain throughout early embryonic life, revealing that it has a pivotal role in neurological development [7]. This appears to justify the disease’s non-specific neurodevelopmental disorder rather than having a specific phenotypic pattern. Most infants with this syndrome exhibit hypotonia or structural abnormalities, but they are not limited to this, as facial dysmorphia and sleep apnea have also been related [1]. The importance of the description of the syndrome lies in its potential for expansion and variety, which is not yet fully defined, making pre-surgical planning for patients with this condition even more challenging and important [8].

**Figure 2:**
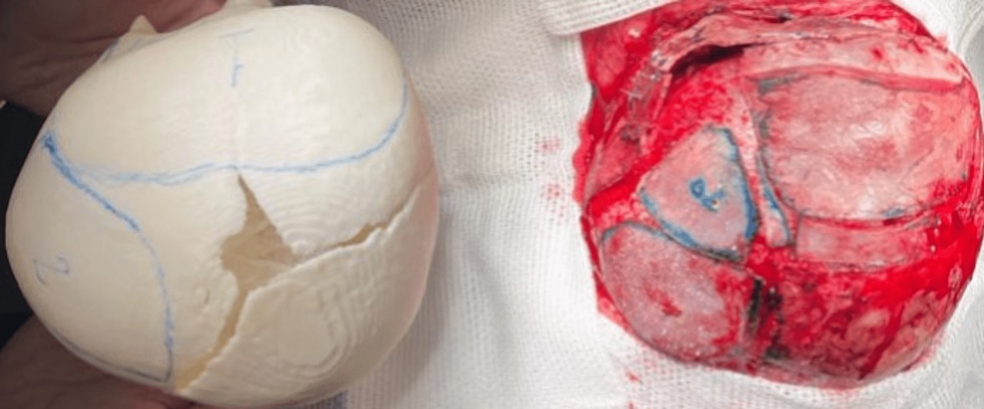
Original photo of the comparison of a cranial vault three-dimensional model and the patient's already surgically remodeled skull

**Figure 3:**
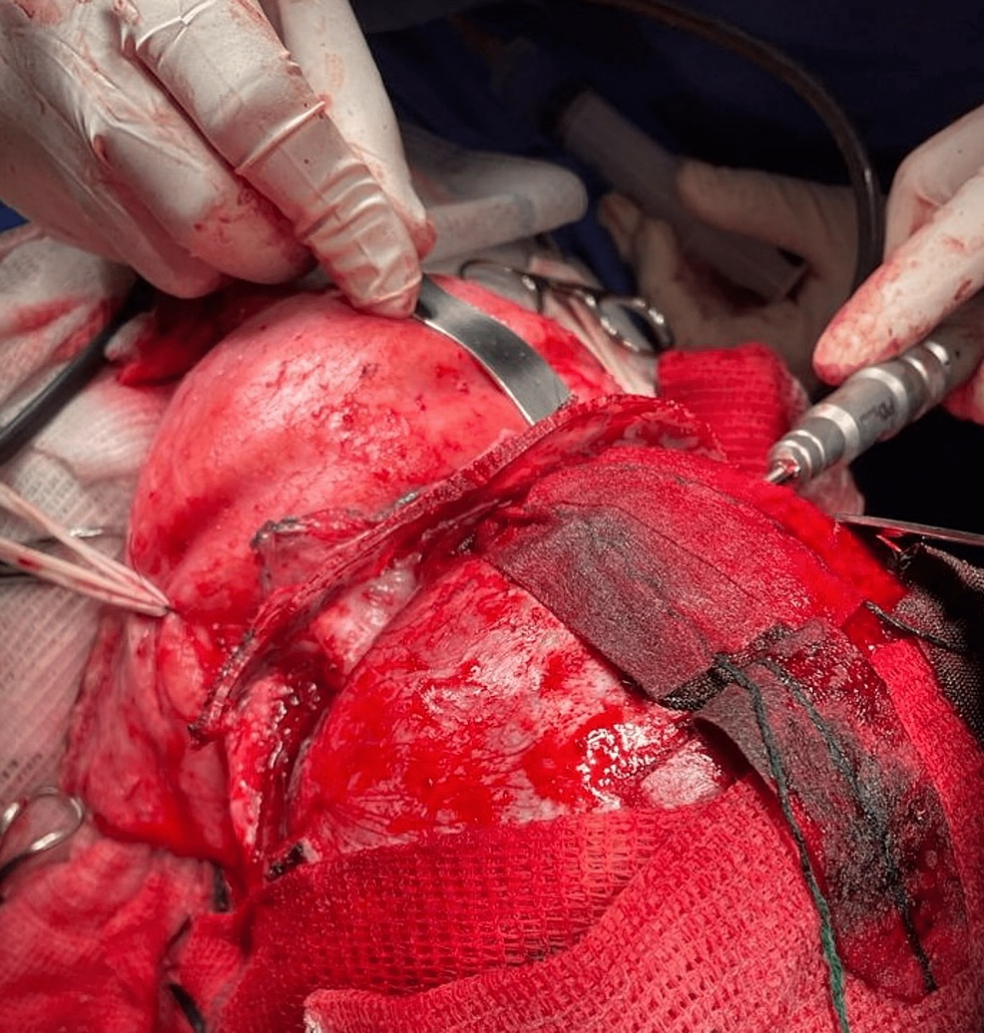
Calvarial vault remodeling with fronto-orbital advancement on the patient

Craniosynostosis is a disorder in which one or more of the cranial sutures fuse prematurely, resulting in abnormal skull development and head shape. At least 80% of cases are associated with syndromic children, with an incidence of about one in every 2500 live births. Treatment is primarily surgical and is determined by the child's age, related comorbidities, and the type of synostosis present [9]. The main one in this case was the left coronal. The chosen surgical technique was calvarial vault remodeling, which consists of openly reshaping cranial sutures based on studies made on a three-dimensional model (Figure 2). Additionally, fronto-orbital advancement had to be done, which means pulling the forehead and eyebrow region forward for better frontal assessment (Figure 3).

Anesthesia management and complications might be challenging in those cases, and their level of difficulty may be associated with the type of surgery. Possible complications included in this case: blood loss requiring moderate to massive transfusions, metabolic disturbances, positional injuries, venous air embolism, oculocardiac reflex, hypothermia, coagulopathies, and airway edema [10]. Due to this and the chosen surgical techniques, we decided to transfuse early, rigorously control volemic reposition, warm, and closely monitor gasometry and hemodynamics.

Our patient was also allergic to both pineapple and latex, resulting in a syndrome characterized by latex and plant-derived food cross-reactions. It includes IgE-mediated symptoms ranging from nasal congestion, mild erythema, and urticaria to anaphylactic shock [11]. Because natural rubber latex is used in the manufacturing of a wide spectrum of medical products, detecting the syndrome during the preoperative evaluation is crucial for developing an efficient surgery and anesthetic plan [12]. Our institute protocol was followed meticulously to provide latex-free surgery and avoid any further complications.

## Conclusions

We presented a patient with a rare disease who underwent elective surgery to repair a left coronal craniosynostosis, with additional challenges due to the coexistence of the latex-fruit syndrome. The procedure was successful with no complications, and our anesthesia management has shown satisfactory results. Among the obstacles that were found, the scarcity of documents in the literature describing anesthetic experiences with the XGS was certainly the most worrisome. In this context, much more has to be studied about this reported rare condition, particularly in the anesthetic scenario.

## References

[1] Xia F, Bainbridge MN, Tan TY (2014). De novo truncating mutations in AHDC1 in individuals with syndromic expressive language delay, hypotonia, and sleep apnea. Am J Hum Genet.

[2] (2023). Xia-Gibbs Syndrome. https://xia-gibbs.org/xia-gibbs-syndrome.

[3] Dey S, Hu Y, Torres GJ, Atoot A, Bangolo A (2023). Jackfruit anaphylaxis due to cross reactivity with latex. J Community Hosp Intern Med Perspect.

[4] Murao S, Nakata H, Roberts I, Yamakawa K (2021). Effect of tranexamic acid on thrombotic events and seizures in bleeding patients: a systematic review and meta-analysis. Crit Care.

[5] Stricker PA, Zuppa AF, Fiadjoe JE (2013). Population pharmacokinetics of epsilon-aminocaproic acid in infants undergoing craniofacial reconstruction surgery. Br J Anaesth.

[6] Mathew A, Rai E (2021). Pediatric perioperative fluid management. Saudi J Anaesth.

[7] Quintero-Rivera F, Xi QJ, Keppler-Noreuil KM (2015). MATR3 disruption in human and mouse associated with bicuspid aortic valve, aortic coarctation and patent ductus arteriosus. Hum Mol Genet.

[8] Cardoso-Dos-Santos AC, Oliveira Silva T, Silveira Faccini A (2020). Novel AHDC1 gene mutation in a Brazilian individual: implications of Xia-Gibbs syndrome. Mol Syndromol.

[9] Pearson A, Matava CT (2016). Anesthetic management for craniosynostosis repair in children. BJA Educ.

[10] Hsieh H, Thornton L, Mann G (2018). Craniosynostosis and anesthetic management for cranial vault remodeling. Neuroanesthesia: A Problem-Based Learning Approach.

[11] Blanco Guerra C (2002). Latex-fruit-syndrome. Allergol Immunopathol.

[12] Parisi CA, Kelly KJ, Ansotegui IJ (2021). Update on latex allergy: new insights into an old problem. World Allergy Organ J.

